# Study of *CaDreb2c* and *CaDreb2h* Gene Sequences and Expression in Chickpea (*Cicer arietinum* L.) Cultivars Growing in Northern Kazakhstan under Drought

**DOI:** 10.3390/plants13152066

**Published:** 2024-07-26

**Authors:** Konstantin V. Kiselev, Zlata V. Ogneva, Alexandra S. Dubrovina, Ademi Zh. Gabdola, Gulmira Zh. Khassanova, Satyvaldy A. Jatayev

**Affiliations:** 1 Laboratory of Biotechnology, Federal Scientific Center of the East Asia Terrestrial Biodiversity, Far Eastern Branch of the Russian Academy of Sciences, Vladivostok 690022, Russia; zlata.v.ogneva@gmail.com (Z.V.O.); dubrovina@biosoil.ru (A.S.D.); 2 Faculty of Agronomy, S. Seifullin Kazakh Agro Technical Research University, Astana 010000, Kazakhstan; for_work_15@mail.ru (A.Z.G.); khasanova-gulmira@mail.ru (G.Z.K.); satidjo@gmail.com (S.A.J.); 3 A.I. Barayev Research and Production Centre of Grain Farming, Shortandy 021600, Kazakhstan

**Keywords:** abiotic stress, crop, gene expression, tolerance, transcription factors

## Abstract

Drought poses a significant challenge to plant growth and productivity, particularly in arid regions like northern Kazakhstan. Dehydration-responsive element-binding (DREB) transcription factors play an important role in plant response to drought and other abiotic stresses. In *Arabidopsis thaliana*, the DREB subfamily consists of six groups, designated DREB1 to DREB6. Among these, DREB2 is primarily associated with drought and salinity tolerance. In the chickpea genome, two *DREB* genes, *CaDREB2c* and *CaDREB2h*, have been identified, exhibiting high sequence similarity to Arabidopsis *DREB2* genes. We investigated the nucleotide sequences of *CaDREB2c* and *CaDREB2h* genes in several chickpea cultivars commonly grown in northern Kazakhstan. Interestingly, the *CaDREB2h* gene sequence was identical across all varieties and corresponded to the sequence deposited in the GenBank. However, the *CaDREB2c* gene sequence exhibited variations among the studied varieties, categorized into three groups: the first group (I), comprising 20 cultivars, contained a *CaDREB2c* gene sequence identical to the GenBank (Indian cultivar CDC Frontier). The second group (II), consisting of 4 cultivars, had a single synonymous substitution (T to C) compared to the deposited *CaDREB2c* gene sequence. The third group, encompassing 5 cultivars, displayed one synonymous substitution (C to T) and two non-synonymous substitutions (G to T and G to A). Furthermore, we assessed the gene expression patterns of *CaDREB2c* and *CaDREB2h* in different chickpea varieties under drought conditions. Chickpea cultivars 8 (III), 37 (I), 6 (III), and 43 (I) exhibited the highest drought resistance. Our analysis revealed a strong positive correlation between drought resistance and *CaDREB2h* gene expression under drought stress. Our findings suggest that the chickpea’s adaptive responses to water deprivation are associated with changes in *CaDREB2* gene expression. To further elucidate the mechanisms underlying drought tolerance, we propose future research directions that will delve into the molecular interactions and downstream targets of *CaDREB2* genes.

## 1. Introduction

Chickpeas (*Cicer arietinum* L.), a globally important legume, rank third in terms of production. Cultivated across 14.84 million hectares, they yield approximately 15.08 million tons annually (FAOSTAT, 2020) [1]. Predominantly grown in the Mediterranean basin, Central Asia, East Africa, Europe, and North and South America, chickpeas serve as a dietary staple due to their rich protein and carbohydrate content [2]. 

Chickpeas are a nutritional powerhouse, providing essential amino acids with high bioavailability, making them a superior protein source among legumes. Additionally, they are gaining recognition for their dietary peptides, which contribute to their overall nutritional value [3]. Beyond their nutritional significance, chickpeas also play a crucial role in crop rotation. Their ability to protect crops from diseases and their nitrogen-fixing properties enhance soil fertility. This nitrogen-fixing ability also has environmental benefits, reducing greenhouse gas emissions, particularly nitrous oxide, a potent greenhouse gas [4]. Despite their adaptability to arid and semi-arid regions, drought remains a significant yield-limiting factor, second only to diseases. Chickpeas are used for food purposes and as animal feed since they contain a significant amount of carbohydrates, all essential amino acids except sulfur-containing types, linoleic and oleic acids, riboflavin, thiamine, folic acid, etc. [5].

Researchers are actively exploring various strategies to improve drought tolerance in chickpeas, including developing drought-resistant varieties and implementing water-saving irrigation practices. Chickpeas are a globally important legume with a range of nutritional and agricultural benefits. Further research and innovation in cultivation practices and drought tolerance will contribute to ensuring a sustainable and reliable supply of this valuable crop [6].

Dehydration-responsive element-binding (DREB) transcription factors are pivotal regulators of plant growth, development, and stress responses. Six groups (DREB1 to DREB6) of DREB subfamily members exist in Arabidopsis thaliana, with A-1 and A-2 being the most studied [7]. The A-1 member *AtCBF1*, induced by low temperatures, positively regulates low-temperature stress responses, as do *AtDREB1A* and *AtDREB1C* [8,9]. *SwDREB1* from sweet potato *Ipomoea batatas* and *ZjDREB1.4* from zoysia grass *Zoysia japonica* also enhance tolerance to low temperatures. In rice, *OsDREB1A*, *OsDREB1B*, and *OsDREB1C* interact with the GCC-box of the promoter region of the target genes to enhance cold tolerance [10,11]. In contrast to DREB1’s role in cold stress, DREB2 is primarily associated with drought and salinity tolerance [12]. *AtDREB2A* and *AtDREB2B*, the initial A-2 members, are induced by dehydration and salinity [7]. Overexpression of soybean *GmDREB2* in Arabidopsis improves salinity tolerance without growth retardation [13]. Heterologous overexpression of sugarcane *EaDREB2* enhances drought and salinity stress tolerance. Research has expanded to other plant species, revealing diverse DREB functions. StDREB from potato (*Solanum tuberosum*) confers drought and salt tolerance in transgenic Arabidopsis [14]. Overexpression of *GhDREB3* from cotton (*Gossypium hirsutum*) improved drought and salt tolerance in transgenic Arabidopsis [15]. 

Moreover, DREB TFs have been implicated in regulating plant responses to heat stress. *AtDREB2A* and *AtDREB2B* expression increases under heat stress, and their overexpression in Arabidopsis enhances heat tolerance [7]. These findings underscore the crucial role of DREB TFs in mediating plant responses to various abiotic stresses, highlighting their potential for developing stress-tolerant crops through genetic engineering approaches. In sum, DREB2 is mainly associated with drought and salinity tolerance [16]. *AtDREB2A* and *AtDREB2B*, the first reported A-2 members, are induced by dehydration and salinity [7]. Therefore, the purpose of this work was to study the involvement of chickpea *DREB2* genes in chickpea resistance to drought.

## 2. Results and Discussion

### 2.1. Selection of Chickpea Cultivars for Drought Experiments Based on Duration of the Growing Season and Crop Yield 

In 2022, 156 samples of different cultivars of chickpeas grown in the north of Kazakhstan were planted: 112 of them were hybrids, and the remaining 44 varieties were of different origins (Table S1). In our work, we decided to use 29 cultivars with the best crop yield and duration of the growing season: cultivars 2, 4, 6, 8, 10, 18, 20, 30, 35, 37, 43, 48, 52, 53, 57, 58, 64, 68, 73, 74, 75, 77, 94, 95, 114, 117, 118, 119, and 120 (Tables S1 and S2).

An extensive study was conducted on 156 varieties of beans, evaluating their growth and productive characteristics. Detailed data on each variety, including plant height, attachment height of the lower bean, weight of 1000 seeds, and seed yield per plant, were meticulously recorded and presented in a supplementary table (Table S1). To narrow down the field for further molecular biological research, a selection process was implemented, focusing on varieties that exhibited superior performance across key parameters. A total of 29 varieties met these criteria and were selected for subsequent molecular studies.

**Figure 1:**
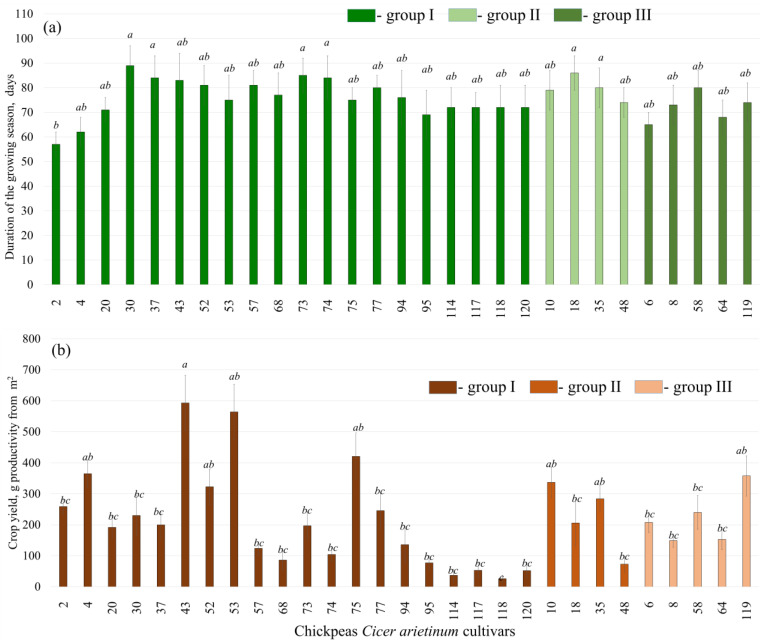
Duration of the growing season in days (**a**) and crop yield in g productivity from the square meter (**b**) of the different cultivars of chickpeas (*Cicer arietinum*). Means followed by the same letter were not different using one-way analysis of variance (ANOVA), followed by the Tukey HSD multiple comparison test (*p* < 0.05). The sequence of chickpea cultivars is presented according to the difference in the sequence of the *CaDreb2c* gene, groups I, II, and III (Figure 2).

Among the selected varieties, cultivars 2, 4, 6, 95, and 64 had the shortest duration of the growing season: less than 70 days (Figure 1a). In other cultivars, this parameter was from 70 to 85 days (Figure 1a). Among the studied chickpea varieties, varieties 4, 10, 53, 119, 75, 52, and 43 exhibited exceptional performance, yielding between 323 and 593 g per m^2^ (g/m^2^) (Figure 1b) or 3.24 and 5.94 tons per hectare (t/ha). These yields are remarkable, as chickpea yields above 2 t/ha are considered high. 

In comparison, studies conducted in various regions of Russia have reported maximum yields of 2.8 t/ha, while the average yield typically ranges from 1 to 1.5 t/ha [17,18]. On the other hand, certain varieties exhibited low crop yields: cultivars 48, 114, 118, 95, 68, 120, and 117 produced less than 100 g/m^2^ (Figure 1b), which translates to less than 1 t/ha. These results highlight the variability in yield performance among different chickpea varieties, emphasizing the importance of selecting suitable varieties adapted to specific growing conditions to maximize productivity.

### 2.2. CaDreb2c and CaDreb2h Gene Sequences in the Different Used Chickpea C. arietinum Cultivars 

We used the CTAB-spin method to extract DNA from 29 different cultivars of chickpeas with shortest duration of the growing season and highest crop yield (Figure 1). Next using specific primers designed for the beginning and the end of the protein-coding region of the *CaDreb2c* and *CaDreb2h* genes we performed PCR analysis on the extracted DNA. 

**Figure 2:**
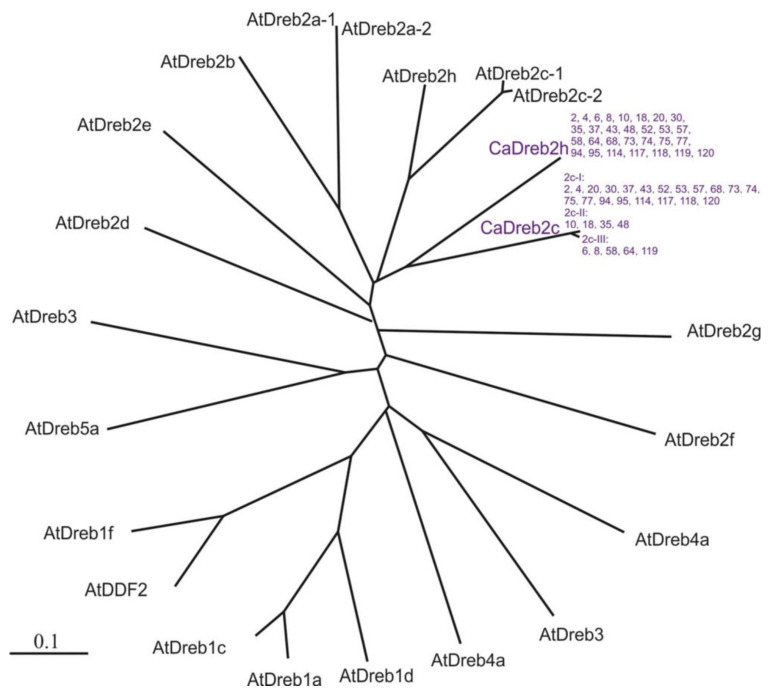
Amino acid sequence radial tree of the chickpeas *Cicer arietinum CaDreb2h* (XM_028371180) and *CaDreb2c* (XM_028361794) genes and all known 20 *Arabidopsis thaliana DREB* genes. AtDREB4A, NM_106369.1; AtDREB5A, NM_105820.3; AtDREB3, NM_127898.4; AtDreb2b, NM_111939.3; AtDreb1a, NM_118680; AtDreb1c, NM_118679.1; AtDREB2F, NM_115620.1; AtDDF2, NM_104981.3; AtDREB4A, NM_101133.2; AtDREB1F, NM_101131.3; AtDREB2H, NM_129595.2; AtDREB2D, NM_106202.2; AtDREB2c-1, NM_001336833; AtDREB2c-2, NM_129594.2; AtDREB2E, NM_129390.2; AtDREB3, NM_121197.2; AtDREB2a-1, NM_120623.3; AtDREB2a-2, NM_120623.3; AtDREB1D, NM_124578.1; AtDREB2G, NM_121850.1 [20]. Multiple sequence alignments and a phylogram based on pairwise alignment were conducted with the ClustalX program 1.81 [21]. The branch lengths are proportional to divergence, with the scale of “0.1” representing a 10% change. Numbers are the chickpea cultivars used in the work (Table S1).

We conducted direct sequencing of the 576 bp PCR products (only coding region; there are no introns in the gene structure) from the *CaDreb2h* gene in all 29 chickpea cultivars. The nucleotide and amino acid sequences of these samples showed no differences (Figure 2). They were identical to the previously deposited sequence of the *CaDreb2h* gene in chickpeas (XM_004487302.3, Indian cultivar CDC Frontier). This information suggests that the analyzed cultivars share the same genetic sequence for the *CaDreb2h* gene. Further research could explore the implications of this genetic similarity on the traits and characteristics of these chickpea cultivars.

More interesting results were obtained by sequencing the 1215 bp PCR products (only coding region; there are no introns in the gene structure) of the *CaDreb2c* gene (Figure 2).

**Figure 3:**
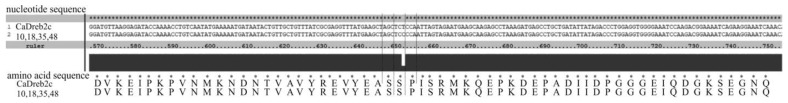
Partial nucleotide sequence of *CaDreb2c* gene of deposited c in GenBank sequence (XM_012713080.2) and sequences from 10, 18, 35, and 48 cultivars (group II) of chickpeas (*Cicer arietinum*). Group II sequence differed by one synonymous substitution (T on C, 651 bp) from XM_012713080.2. * indicates the same positions of nucleotides or amino acids in sequences.

**Figure 4:**
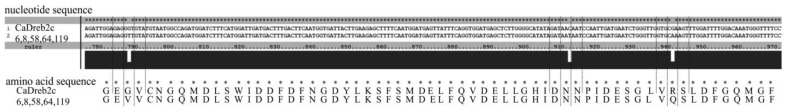
Partial nucleotide sequence of *CaDreb2c* gene of deposited c in GenBank sequence (XM_012713080.2) and sequences from 6, 58, 64, and 119 cultivars (group III) of chickpeas (*Cicer arietinum*). Group III sequence differed one synonymous substitution (C on T, 910 bp) and two non-synonymous substitution (G on T, 790 bp, substitution aminoacid glycine on valine; G on A, 940 bp, substitution aminoacid arginine on glutamine) from the deposited in GenBank *CaDreb2c* gene sequence (#6, 8, 58, 64, and 119). * indicates the same positions of nucleotides or amino acids in sequences.

All sequences were divided into three groups (Figure 3 and Figure 4):

- Twenty were identical for deposited c in GenBank chickpeas *CaDreb2c* gene sequence (XM_012713080.2, Indian cultivar CDC Frontier) (#2, 4, 20, 30, 37, 43, 52, 53, 57, 68, 73, 74, 75, 77, 94, 95, 114, 117, 118, and 120).
- Four differed by one synonymous substitution (T on C, 651 bp) from the deposited *CaDreb2c* gene sequence of cultivar CDC Frontier (#10, 18, 35, and 48) (Figure 3).
- Five differed by one synonymous substitution (C on T, 910 bp) and two non-synonymous substitutions (G on T, 790 bp; G on A, 940 bp) from the deposited in GenBank *CaDreb2c* gene sequence of cultivar CDC Frontier (#6, 8, 58, 64, and 119) (Figure 4). Using a search for conserved domains within a protein sequence (CD-search, National Center for Biotechnology Information, NCBI, https://www.ncbi.nlm.nih.gov, accessed on 23 July 2024), we found DNA-binding domains in plant proteins such as APETALA2 and EREBPs [19]. This domain is located closer to the beginning of the gene (interval 85–145 aa, E-value 3.74 × 10^−34^). Thus, the found substitutions do not change the structure of the main DNA-binding domain.

Moreover, Figure 2 proves once again that our *CaDreb2c* and *CaDreb2h* genes were the closest to the *AtDreb2c* and *AtDreb2h* genes of Arabidopsis. Next, it would be intriguing to uncover the source of the origin of the examined *CaDREB2c* sequences. These data have been displayed in Table S2. Overall, there is no definitive correlation between the *CaDREB2c* gene sequence and the geographical region of origin. However, it is worth noting that the *CaDREB2c* III group was predominantly of Russian and Iranian cultivars (Table S2). There is only the I form in the GenBank, so we first described the II and III forms.

### 2.3. Chickpeas C. arietinum Cultivar Resistance to Drought in Experimental Conditions

**Figure 5:**
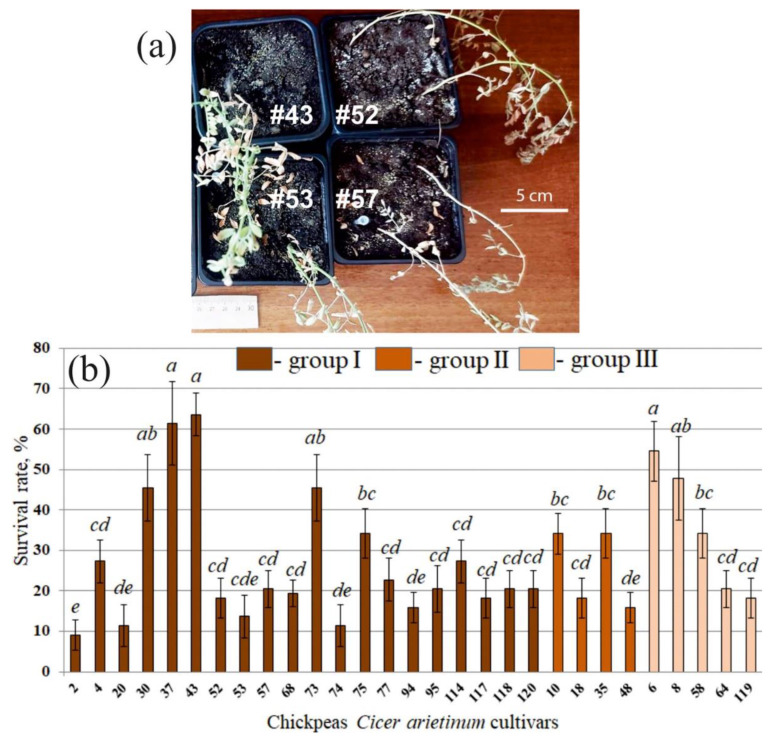
Impact of drought on the survival rate of the chickpea cultivars (*Cicer arietinum*). The data were collected from seven independent experiments, each consisting of two pots. Within each pot, there were two chickpea plants (**a**). To induce drought stress, the plants were cultivated without water for 4 weeks. After this period, the plants were watered again, and the number of surviving green plants was counted after 5 days to determine the survival rate (**b**). Means followed by the same letter were not different using one-way analysis of variance (ANOVA), followed by the Tukey HSD multiple comparison test (*p* < 0.05). The sequence of chickpea cultivars is presented according to the difference in the sequence of the *CaDreb2c* gene, groups I, II, and III (Figure 2).

We used experimental drought conditions to investigate the resistance of the studied cultivars of chickpeas *C. arietinum* to drought. To do this, we cultivated chickpea seedlings for two weeks, followed by withholding watering for another two weeks. Then, all the pots were filled with water, and five days later, the survival rate of the plants was assessed. The surviving plants showed signs of recovery, displaying healthy green foliage. In contrast, the plants that had succumbed to the drought lay on the ground with dried, yellow leaves (Figure 5a).

Using experimental drought conditions, we showed that cultivars 43 (I), 37 (I), 6 (III), 8 (III), 30 (I), and 73 (I) had the highest survival rate after drought (strong, resistant group): more than 45% of all plants were green; those varieties showed 45–65% of surviving plants after drought experiments (Figure 5b). At the same time, the average resistance value of all 29 chickpea cultivars was 27.7 ± 0.4%. Thus, the strong, resistant group was 1.7–2.3 times more resistant to drought than the average survival rate of the used 29 cultivars. Moreover, statistical treatment showed that the survival rates of these varieties (43, 37, 6, 8, 30, 73) were significantly higher than in 19 other varieties from 23 remaining cultivars. Also, cultivars 2, 20, 53, 74, 94, and 48 withstood this abiotic stress the worst—less than 16% (Figure 5b).

It is interesting to note that four out of six drought-resistant and five out of six drought-sensitive varieties belonged to group I according to the sequence of the *CaDreb2c* gene. There were no strong resistant varieties in the second (II) group. Group III was the most interesting because among only five varieties, two cultivars were drought-resistant, and there were no drought-sensitive varieties. 

Moreover, chickpea cultivars 6 (III), 8 (III), 37 (I), and 43 (I) exhibited the highest drought resistance. Thus, among the 29 studied chickpea cultivars, 4 were the most consistently resistant to drought, and 2 of them belonged to the III group according to the *CaDreb2c* gene. Furthermore, we assessed the gene expression patterns of *CaDREB2c* and *CaDREB2h* in different chickpea varieties under control and drought conditions. 

### 2.4. CaDreb2c and CaDreb2h Gene Expression in the Different Used Chickpea C. arietinum Cultivars in Normal and Drought Conditions

To study the gene expression of *CaDreb2c* and *CaDreb2h* quantitatively, we conducted real-time quantitative PCR (RT-qPCR) on cDNA probes derived from two-week-old seedlings and four-week-old plants that were subjected to drought stress without watering. We focused on analyzing the gene expression of *CaDreb2c* and *CaDreb2h* in 29 different chickpea *C. arietinum* cultivars under drought-stress conditions in four-week-old plants. 

**Figure 6:**
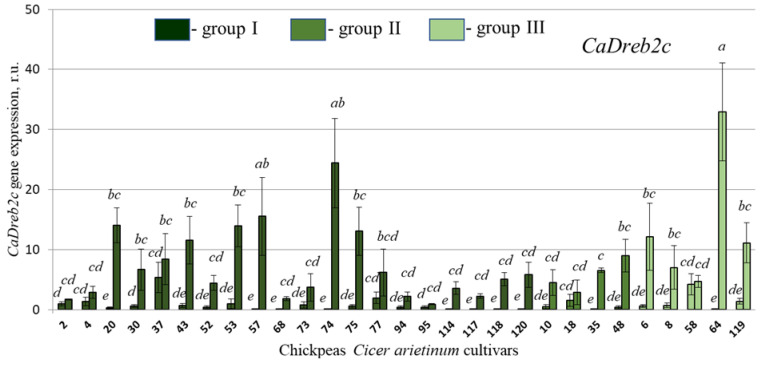
Quantification of the *CaDreb2c* expression levels in the chickpeas *Cicer arietinum* cultivars performed by quantitative RT-qPCR before (left column) and after drought stress (right column). RNA was extracted from two-week seedlings (left column) and four-week plants growing without watering under drought stress (right column). Statistical analysis for experimental data is typically reported as mean values along with the standard error (SE). In this case, four independent experiments were conducted, each with ten technical replicates (five for *CaGAPDH*, five for *CaEF*). Means followed by the same letter were not different using one-way analysis of variance (ANOVA), followed by the Tukey HSD multiple comparison test (*p* < 0.05). The sequence of chickpea cultivars is presented according to the difference in the sequence of the *CaDreb2c* gene, groups I, II, and III (Figure 2).

**Figure 7:**
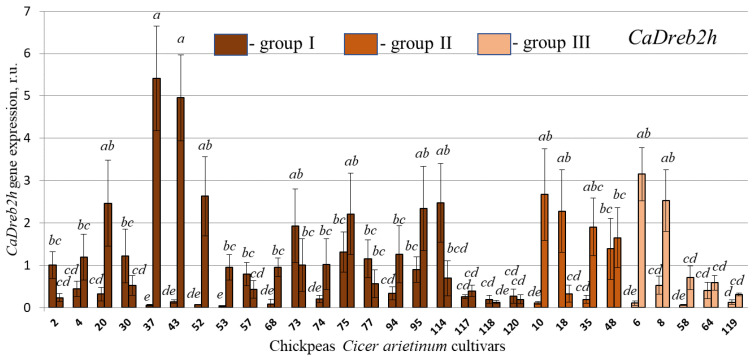
Quantification of the *CaDreb2h* expression levels in the chickpeas *Cicer arietinum* cultivars performed by quantitative RT-qPCR before and after drought stress. RNA was extracted from two-week seedlings and four-week plants growing without watering under drought stress. Statistical analysis for experimental data is typically reported as mean values along with the standard error (SE). In this case, four independent experiments were conducted, each with ten technical replicates (five for *CaGAPDH*, five for *CaEF*). Means followed by the same letter were not different using one-way analysis of variance (ANOVA), followed by the Tukey HSD multiple comparison test (*p* < 0.05). The sequence of chickpea cultivars is presented according to the difference in the sequence of the *CaDreb2c* gene, groups I, II, and III (Figure 2).

The results indicated that the expression of these genes was higher in the four-week-old plants experiencing drought stress compared to the two-week-old seedlings (Figure 6 and Figure 7). These data suggest that these genes play an essential role in raising the plant’s ability to tolerate drought conditions, which confirms the previously obtained data [7], although, of course, 2 weeks of plant cultivation can also have some effect on *CaDreb2c* and *CaDreb2h* expression.

Normally, before the drought, in 2-week chickpea seedlings, the expression of the *CaDreb2c* gene was highest in cultivars 37 (I), 77 (I), 18 (II), 58 (III), and 119 (III) (Figure 6) and the *CaDreb2h* gene in cultivars 2 (I), 30 (I), 73 (I), 75 (I), 77 (I), 114 (I), 18 (II), and 48 (II) (Figure 7). However, it was more important to test the expression of these genes under drought. Drought significantly increased the expression of the *CaDreb2c* and *CaDreb2h* genes in many of the studied chickpea cultivars (Figure 6 and Figure 7), which partly proves the positive participation of these genes in plant resistance to drought.

It has been shown that under the influence of drought, the expression of the *CaDreb2c* gene increased in 20, 30, 43, 53, 57, 74, 75, 114, 118, 120, 35, 48, 6, 8, 64, and 119 cultivars (Figure 6). Also, under the influence of drought, the expression of the *CaDreb2h* gene increased in cultivars 20, 37, 43, 52, 53, 68, 10, 6, and 8 (Figure 7).

Next, we performed a correlation analysis between drought resistance (Figure 5b) and the expression of the *CaDREB2c* and *CaDREB2h* genes before and after drought treatment (Figure 6 and Figure 7). We showed a small positive correlation (r = 0.40) between drought resistance (Figure 5b) and the expression of the *CaDREB2c* gene before drought (Figure 6). Perhaps without drought, high *CaDREB2c* gene expression contributes to preparing and accumulating the necessary protective molecules for drought. Moreover, after exposure to drought, *CaDREB2c* expression increased, but this increase was not correlated (r = −0.05) with drought resistance (Figure 5b). 

However, a stronger positive correlation was observed between drought resistance and *CaDREB2h* gene expression under drought stress (r = 0.67 with *p* = 0.002, Figure 7) and enhancing the level of the *CaDREB2h* gene expression under drought stress (r = 0.54 with *p* = 0.003, Figure 7). 

## 3. Materials and Methods

### 3.1. Plant Material 

The research was carried out in 2022 on the basis of the experimental agricultural breeding station of the Kazakh Agrotechnical Research University named after S. Seifullin, located in the dry-steppe zone of Northern Kazakhstan (Akmola region, Shortandinsky district). The soil of the experimental station is southern carbonate chernozem. We used a field that has not been sown for at least one year.

### 3.2. Drought Stress Experiments

Seeds of chickpeas were germinated 5 days on filter paper moistened with sterile filtered water. The seedlings were then transferred to commercially available well-watered rich soil in a controlled environmental chamber at 22 °C (KBW 400, Binder, Tuttlingen, Germany) kept on a 16/8 h day/night cycle at a light intensity of approximately 120 μmol m^−2^s^−1^.

The chickpea plants were exposed to drought stress, as described earlier [22]. We cultivated chickpea seedlings for two weeks, followed by withholding watering for another two weeks. By the end of this time frame, the impact of the drought was evident on the majority of the plants, as they wilted and exhibited yellowing of some leaves. Subsequently, all the containers housing the 4-week-old chickpea plants were watered, and five days later, the survival rate of the plants was assessed. The surviving plants showed signs of recovery, displaying healthy green foliage. In contrast, the plants that had succumbed to the drought lay on the ground with dried, yellow leaves. Survival rates were determined by counting the number of visibly green chickpea plants five days after watering [22].

### 3.3. Nucleic Acid Purification and Real-Time Quantitative PCR (RT-qPCR)

The cetyltrimethylammonium bromide (CTAB) based extraction method was utilized to isolate total DNA as outlined in previous studies [23]. This extraction technique effectively isolates DNA from plant samples, including those with complex genomic structures [22]. The CTAB-based extraction method was employed for total RNA isolation, as described [24]. This approach ensures high-quality RNA extraction, preserving the integrity of the RNA molecules [23]. The isolated RNA was then used for cDNA synthesis using the MMLV Reverse transcription PCR Kit with oligo(dT)15 (RT-PCR, Evrogen, Moscow, Russia) as described [24].

The mRNA transcript levels in RT-qPCR of the *CaDREB2c* and *CaDREB2h* genes were determined by the 2^−ΔΔCT^ method [25]. This method involves comparing the expression of the target genes to internal control, such as glyceraldehyde 3-phosphate dehydrogenase (*CaGAPDH*, NM_001365164.1) and elongation factor (*CaEF*, AJ004960.1) [26]. The primers designed for RT-qPCRs are provided in Supplementary Table S3. RT-qPCR reactions were performed in volumes of 20 µL using the real-time PCR kit (Evrogen, Moscow, Russia [27]) and contained 1 × Taq buffer, 2.5 mM MgCl_2_, 0.2 mM of each dNTP, 0.2 µM of each primer, 1 × SybrGreen I real-time PCR dye, 1 µL cDNAs, and 1 unit of Taq DNA polymerase (Evrogen). The reaction was conducted in a DTprime 4M1 Thermal Cycler (DNA-technology, Moscow, Russia) using the following cycling parameters: initial denaturation at 95 °C for 2 min, followed by 60 cycles of 10 s at 95 °C and 25 s at 62 °C [28]. This optimized RT-qPCR protocol allows for accurate and sensitive detection of gene expression levels, providing valuable insights into the molecular mechanisms underlying transgene expression and regulation in plants.

### 3.4. Statistical Analysis

The data are shown as mean ± standard error (SE) and were evaluated by paired Student’s *t*-test. The *p* < 0.05 level was selected as the point of minimal statistical significance in all analyses, where *p* < 0.05 was considered to be statistically significant. The Pearson correlation coefficient (r and *p*-value) was calculated in Microsoft Excel Standard 2019 (Microsoft Office, Microsoft, Redmond, WA, USA).

## 4. Conclusions

In summary, the data obtained revealed that the selection process aimed to identify chickpeas *C. arietinum* cultivars with desirable traits that could potentially contribute to improved bean production and resilience. By focusing on cultivars that excel in key agronomic characteristics, researchers can gain valuable insights into the genetic basis of these traits and potentially develop improved chickpea varieties with enhanced performance and adaptability. 

The main task is to find the genes that are responsible for resistance to the adverse growing conditions of interest. For example, dehydration-responsive element-binding transcription factors play a critical role in plant response to drought and other abiotic stresses. We revealed a weak positive correlation between drought resistance and *CaDREB2c* gene expression in control conditions before the drought experiments. Perhaps without drought, high *CaDREB2c* gene expression contributes to preparing and accumulating the necessary protective molecules for drought. Moreover, the III cultivars group based on the gene *CaDREB2c* sequence was the most interesting for biotechnology because among only five varieties, two cultivars were drought-resistant, and there were no drought-sensitive varieties. Also, a stronger positive correlation was observed between drought resistance and *CaDREB2h* gene expression under drought stress. 

Our research indicates that chickpea’s ability to adapt to water scarcity is linked to alterations in the expression of the *DREB2* genes. This suggests the potential use of the CaDREB2c and CaDREB2h genes in the selection of chickpea cultivars that are more tolerant to drought. Further research is needed to elucidate the specific mechanisms underlying the differential expression of *CaDREB2* genes and their contribution to drought resistance in chickpeas. It is now evident that *DREB2* genes function as transcription factors, as evidenced by the presence of a DNA binding domain, and they are involved in activating the expression of specific protective genes, e.g., genes of the biosynthesis of osmolytes, antioxidants, dehydrins, ion transporters, etc. This research has the potential to guide future crop improvement efforts, enabling the development of drought-resilient chickpea varieties that can thrive even in challenging environmental conditions, ensuring food security and agricultural sustainability in water-scarce regions.

## Data Availability

The data presented in this study are available in the article and in Supplementary Materials.

## Supplementary Materials

The following supporting information can be downloaded at https://www.mdpi.com/article/10.3390/plants13152066/s1, Table S1. One hundred fifty-six samples of different cultivars of chickpeas grown in the north of Kazakhstan were used in the analysis. Table S2. Geographic origin of the cultivars used in the analyses of the *CaDreb2c* and *CaDreb2h* genes expression. Table S3. Primers used for amplification of chickpea *Cicer arietinum* DNAs and cDNAs of *CaDreb2c* and *CaDreb2h* genes in PCR.
