# Supplementary material for: Study of CaDreb2c and CaDreb2h Gene Sequences and Expression in Chickpea (Cicer arietinum L.) Cultivars Growing in Northern Kazakhstan under Drought
Source: Plants (Basel). 2024 Jul 26;13(15):2066. doi: 10.3390/plants13152066 (PMC11314285; doi:10.3390/plants13152066)
Supplement: Supplementary file 1 [file plants-13-02066-s001.zip › 2024-07b-Cicer-CaDreb2c,h-32suppl.docx]

**Table S2**. Geographic origin of the cultivars used in the analyses of the *CaDreb2c* and *CaDreb2h* genes expression

| № | Group | Cultivars | Hybrids | Geographic origin of the cultivars |
| --- | --- | --- | --- | --- |
| 1 | *CaDreb2c* - I | 2 | G.k.35-ICC 10945хLuch-3-1 | India/Kazakhstan |
| 2 | *CaDreb2c* – I | 4 | G.k.35-ICC 10945хLuch-3-2 | India/Kazakhstan |
| 3 | *CaDreb2c* – I | 20 | Г.к .35-ICC 10945хLuch-15-2 | India/Kazakhstan |
| 4 | *CaDreb2c* – I | 30 | G.k.№36/2-Privo х ІСС 1098 -3 | Russia/Iran |
| 5 | *CaDreb2c* – I | 37 | Г.к .35-ICC 10945хLuch-10 | India/Kazakhstan |
| 6 | *CaDreb2c* - I | 43 | Г.к .23/2-Tassay х ICC 1052-13 | Kazakhstan/Pakistan |
| 7 | *CaDreb2c* - I | 52 | G.k.11-2-ICC 5613 х Kamila -7 | India/Kazakhstan |
| 8 | *CaDreb2c* – I | 53 | G.k.11-2-ICC 5613 х Kamila -1-3 | India/Kazakhstan |
| 9 | *CaDreb2c* – I | 57 | G.k.№36/2-Privo х ІСС 1098 -3 | Russia/Iran |
| 10 | *CaDreb2c* – I | 68 | G.k.11-1-ICC 5613 х Kamila -2 | India/Kazakhstan |
| 11 | *CaDreb2c* – I | 73 | G.k.18/3-3-Krasnokutskaya 123 х ICC 12654-14 | Russia/ Ethiopia |
| 12 | *CaDreb2c* - I | 74 | G.k.18/3-1-Krasnokutskaya 123 х ICC 12654-2 | Russia/ Ethiopia |
| 13 | *CaDreb2c* - I | 75 | G.k.18/3-1-Krasnokutskaya 123 х ICC 12654-1 | Russia/ Ethiopia |
| 14 | *CaDreb2c* – I | 77 | Г.к .30/2-ICC 1083хKamila-15 | Iran/Kazakhstan |
| 15 | *CaDreb2c* – I | 94 | G.k.18/3-1-Krasnokutskaya 123 х ICC 12654-5 | Russia/ Ethiopia |
| 16 | *CaDreb2c* – I | 95 | G.k.18/3-1-Krasnokutskaya 123 х ICC 12654-5 | Russia/ Ethiopia |
| 17 | *CaDreb2c* – I | 114 | G.k.24-349х405 - 28-Б х ICC9590 | Ukraine/ Egypt |
| 18 | *CaDreb2c* - I | 117 | G.k.10-263х118 - ICC9895 х ICC5878 | Afghanistan/India |
| 19 | *CaDreb2c* – I | 118 | G.k.9-256х156 - ICC6306 х ICC2580 | USSR/Iran |
| 20 | *CaDreb2c* – I | 120 | G.k.3-38х3- Lin С 29 х ICC 3325 | Ukraine/Cyprus |
| 21 | *CaDreb2c* - II | 10 | G.k.№36/3-Privo х ІСС 1098 -1-2 | Russia/Iran |
| 22 | *CaDreb2c* – II | 18 | Г.к .23/2-Tassay х ICC 1052-13-1 | Kazakhstan/Pakistan |
| 23 | *CaDreb2c* – II | 35 | Г.к .35-ICC 10945хLuch-14-2 | India/Kazakhstan |
| 24 | *CaDreb2c* - II | 48 | G.k.36/3-♀Privo х ♂ІСС 1098-2-3 | Russia/Iran |
| 25 | *CaDreb2c* - III | 6 | Г.к .41-ІСС 1098 х Privo -2-2 | Iran/Russia |
| 26 | *CaDreb2c* - III | 8 | Г.к .41-ІСС 1098 х Privo -2-1 | Iran/Russia |
| 27 | *CaDreb2c* - III | 58 | G.k.18/3-1-Krasnokutskaya 123 х ICC 12654-1 | Russia/ Ethiopia |
| 28 | *CaDreb2c* - III | 64 | Г.к .41-ІСС 1098 х Privo -2 | Iran/Russia |
| 29 | *CaDreb2c* - III | 119 | G.k.8-252/204 - ICC11284 х ICC9002 | USSR/Iran |

**Table S3.** Primers used for amplification of *chickpea Cicer arietinum* DNAs and cDNAs of *CaDreb2c* and *CaDreb2h* genes in PCR.

| cDNA | Primers names | Primers sequences, 5ꞌ-3ꞌ |
| --- | --- | --- |
| **Primers for obtaining of the full *CaDreb2c and CaDreb2h* gene sequences** | | |
| Full *CaDreb2c* DNA obtaining | CaDreb2c-nachS  CaDreb2c-KonA | 5'ATGGGTGCTGCTTACGAACAA,  5'TTAGATTCCTCTTGAATCATG |
| Full *CaDreb2h* DNA obtaining | CaDreb2h-nachS  CaDreb2h-KonA | 5'ATGATAGTGAAAGCCTGTGAT,  5'CTAATTCACACCTTCCTCATT |
| **Primers for cDNA quality control** | | |
| Cicer arietinum glyceraldehyde-3-phosphate dehydrogenase, cytosolic (GAPDH) | CaGAPDH-OT-s  CaGAPDH-OT-a | 5'ACTCAGAAGACTGTTGATGG,  5'AGTATCACCAATGAAGTCGGT |
| **Primers for real-time PCRs** | | |
| *CaDreb2c* gene | CaDreb2c-real-s  CaDreb2c-real-a | 5'CTTCAAGCTTCTTTTATGAATTGGAAA,  5'TTAGATTCCTCTTGAATCATG |
| *CaDreb2h* gene | CaDreb2h-real-s  CaDreb2h-real-a | 5'TCCGAGTCCATGATAATGCCAA,  5'CTAATTCACACCTTCCTCATT |
| *CaGAPDH* gene | CaGAPDH-real-s  CaGAPDH-real-a | 5'GAAGGCCGCTACCTACGAC  5'AGTATCACCAATGAAGTCGGTG |
|  |  |  |

| 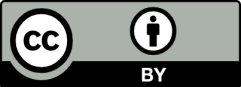 | © 2024 by the authors. Submitted for possible open access publication under the terms and conditions of the Creative Commons Attribution (CC BY) license (http://creativecommons.org/licenses/by/4.0/). |
| --- | --- |
