# Supplementary material for: Study of CaDreb2c and CaDreb2h Gene Sequences and Expression in Chickpea (Cicer arietinum L.) Cultivars Growing in Northern Kazakhstan under Drought
Source: Plants (Basel). 2024 Jul 26;13(15):2066. doi: 10.3390/plants13152066 (PMC11314285; doi:10.3390/plants13152066)
Supplement: Supplementary file 1 [file plants-13-02066-s001.zip › plants-3007970-supplementary/2024-07b-Cicer-CaDreb2c,h-32suppl.pdf]

plants

MDPI

Table S2. Geographic origin of the cultivars used in the analyses of the *CaDreb2c* and *CaDreb2h* genes expression

№	Group	Cultivars	Hybrids	Geographic origin of the cultivars
1	*CaDreb2c* - I	2	G.k.35-ICC 10945xLuch-3-1	India/Kazakhstan
2	*CaDreb2c* – I	4	G.k.35-ICC 10945xLuch-3-2	India/Kazakhstan
3	*CaDreb2c* – I	20	Г.к .35-ICC 10945xLuch-15-2	India/Kazakhstan
4	*CaDreb2c* – I	30	G.k.№36/2-Privo x ICC 1098 -3	Russia/Iran
5	*CaDreb2c* – I	37	Г.к .35-ICC 10945xLuch-10	India/Kazakhstan
6	*CaDreb2c* - I	43	Г.к .23/2-Tassay x ICC 1052-13	Kazakhstan/Pakistan
7	*CaDreb2c* - I	52	G.k.11-2-ICC 5613 x Kamila -7	India/Kazakhstan
8	*CaDreb2c* – I	53	G.k.11-2-ICC 5613 x Kamila -1-3	India/Kazakhstan
9	*CaDreb2c* – I	57	G.k.№36/2-Privo x ICC 1098 -3	Russia/Iran
10	*CaDreb2c* – I	68	G.k.11-1-ICC 5613 x Kamila -2	India/Kazakhstan
11	*CaDreb2c* – I	73	G.k.18/3-3-Krasnokutskaya 123 x ICC 12654-14	Russia/ Ethiopia
12	*CaDreb2c* - I	74	G.k.18/3-1-Krasnokutskaya 123 x ICC 12654-2	Russia/ Ethiopia
13	*CaDreb2c* - I	75	G.k.18/3-1-Krasnokutskaya 123 x ICC 12654-1	Russia/ Ethiopia
14	*CaDreb2c* – I	77	Г.к .30/2-ICC 1083xKamila-15	Iran/Kazakhstan
15	*CaDreb2c* – I	94	G.k.18/3-1-Krasnokutskaya 123 x ICC 12654-5	Russia/ Ethiopia
16	*CaDreb2c* – I	95	G.k.18/3-1-Krasnokutskaya 123 x ICC 12654-5	Russia/ Ethiopia
17	*CaDreb2c* – I	114	G.k.24-349x405 - 28-Б x ICC9590	Ukraine/ Egypt
18	*CaDreb2c* - I	117	G.k.10-263x118 - ICC9895 x ICC5878	Afghanistan/India
19	*CaDreb2c* – I	118	G.k.9-256x156 - ICC6306 x ICC2580	USSR/Iran
20	*CaDreb2c* – I	120	G.k.3-38x3- Lin C 29 x ICC 3325	Ukraine/Cyprus
21	*CaDreb2c* - II	10	G.k.№36/3-Privo x ICC 1098 -1-2	Russia/Iran
22	*CaDreb2c* – II	18	Г.к .23/2-Tassay x ICC 1052-13-1	Kazakhstan/Pakistan
23	*CaDreb2c* – II	35	Г.к .35-ICC 10945xLuch-14-2	India/Kazakhstan
24	*CaDreb2c* - II	48	G.k.36/3- ♀ Privo x ♂ ICC 1098-2-3	Russia/Iran
25	*CaDreb2c* - III	6	Г.к .41-ICC 1098 x Privo -2-2	Iran/Russia
26	*CaDreb2c* - III	8	Г.к .41-ICC 1098 x Privo -2-1	Iran/Russia
27	*CaDreb2c* - III	58	G.k.18/3-1-Krasnokutskaya 123 x ICC 12654-1	Russia/ Ethiopia
28	*CaDreb2c* - III	64	Г.к .41-ICC 1098 x Privo -2	Iran/Russia
29	*CaDreb2c* - III	119	G.k.8-252/204 - ICC11284 x ICC9002	USSR/Iran

Table S3. Primers used for amplification of *chickpea Cicer arietinum* DNAs and cDNAs of *CaDreb2c* and *CaDreb2h* genes in PCR.

cDNA	Primers names	Primers sequences, 5'-3'
**Primers for obtaining of the full *CaDreb2c and CaDreb2h* gene sequences**		
Full *CaDreb2c* DNA obtaining	CaDreb2c-nachS<br>CaDreb2c-KonA	5'ATGGGTGCTGCTTACGAACAA,<br>5'TTAGATTCCTCTTGAATCATG
Full *CaDreb2h* DNA obtaining	CaDreb2h-nachS<br>CaDreb2h-KonA	5'ATGATAGTGAAAGCCTGTGAT,<br>5'CTAATTCACACCTTCCTCATT
**Primers for cDNA quality control**		
Cicer arietinum glyceraldehyde-3-phosphate dehydrogenase, cytosolic (GAPDH)	CaGAPDH-OT-s<br>CaGAPDH-OT-a	5'ACTCAGAAGACTGTTGATGG,<br>5'AGTATCACCAATGAAGTCGGT
**Primers for real-time PCRs**		
CaDreb2c gene	CaDreb2c-real-s<br>CaDreb2c-real-a	5'CTTCAAGCTTCTTTTATGAATTGGAAA,<br>5'TTAGATTCCTCTTGAATCATG
CaDreb2h gene	CaDreb2h-real-s<br>CaDreb2h-real-a	5'TCCGAGTCCATGATAATGCCAA,<br>5'CTAATTCACACCTTCCTCATT
CaGAPDH gene	CaGAPDH-real-s<br>CaGAPDH-real-a	5'GAAGGCCGCTACCTACGAC<br>5'AGTATCACCAATGAAGTCGGTG
